# Predictors of antibiotic prescriptions: a knowledge, attitude and practice survey among physicians in tertiary hospitals in Nigeria

**DOI:** 10.1186/s13756-021-00940-9

**Published:** 2021-04-30

**Authors:** Dimie Ogoina, Garba Iliyasu, Vivian Kwaghe, Akan Otu, Iorhen Ephram Akase, Olukemi Adekanmbi, Dalhat Mahmood, Micheal Iroezindu, Shamsudin Aliyu, Abisoye Sunday Oyeyemi, Stella Rotifa, Mukhtar Abdulmajid Adeiza, Uche Sonny Unigwe, Juliet Ijeoma Mmerem, Farouq Muhammad Dayyab, Zaiyad Garba Habib, Daniel Otokpa, Emmanuel Effa, Abdulrazaq Garba Habib

**Affiliations:** 1 Dimie Ogoina Infectious Disease Unit, Department of Internal Medicine, Niger Delta University/Niger Delta University Teaching Hospital, Yenagoa, Bayelsa State Nigeria; 2 Infectious Disease Unit, Department of Internal Medicine, Aminu Kano University Teaching Hospital, Kano, Nigeria; 3 Infectious Disease Unit, Department of Internal Medicine, University of Abuja Teaching Hospital, Abuja, Gwagwadala, Nigeria; 4 Department of Internal Medicine, College of Medical Sciences, University of Calabar, Calabar, Cross River State Nigeria; 5 Department of Internal Medicine, Lagos State University Teaching Hospital, Lagos, Nigeria; 6 Department of Medicine, University College Hospital/University of Ibadan, Ibadan, Oyo State Nigeria; 7 Nigeria Centre for Disease Control (NCDC), African Field Epidemiology Network (AFENET), Abuja, Nigeria; 8 Department of Medicine, University of Nigeria Teaching Hospital Ituku/Ozalla Enugu, Ituku, Enugu State Nigeria; 9 Department of Medical Microbiology, Ahmadu Bello University Teaching Hospital, Zaria, Kaduna State Nigeria; 10 Department of Community Medicine, Niger Delta University Teaching Hospital, Okolobiri, Bayelsa State Nigeria; 11 Department of Community Medicine, Federal Medical Centre Yenagoa, Yenagoa, Bayelsa State Nigeria; 12 Department of Internal Medicine, Ahmadu Bello University Teaching Hospital, Zaria, Kaduna State Nigeria; 13 Present Address: Infectious Disease Hospital, Kano, Nigeria; 14 Department of Medicine, Federal Medical Center, Nguru, Yobe State Nigeria

**Keywords:** Antimicrobial resistance, KAP, Antibiotic prescriptions, Antimicrobial stewardship, Nigeria

## Abstract

**Background:**

As part of the Global Action Plan against antimicrobial resistance (AMR), countries are required to generate local evidence to inform context-specific implementation of national action plans against AMR (NAPAR). We aimed to evaluate the knowledge, attitude, and practice (KAP) regarding antibiotic prescriptions (APR) and AMR among physicians in tertiary hospitals in Nigeria, and to determine predictors of KAP of APR and AMR.

**Methods:**

In this cross-sectional study, we enrolled physicians practicing in tertiary hospitals from all six geopolitical zones of Nigeria. Implementation of an antimicrobial stewardship programmes (ASP) by each selected hospital were assessed using a 12 item ASP checklist. We used a structured self-administered questionnaire to assess the KAP of APR and AMR. Frequency of prescriptions of 18 different antibiotics in the prior 6 months was assessed using a Likert’s scale. KAP and prescription (Pr) scores were classified as good (score ≥ 80%) or average/poor (score < 80%). Independent predictors of good knowledge, attitude, and practice (KAPPr) were ascertained using an unconditional logistic regression model.

**Results:**

A total of 1324 physicians out of 1778 (74% response rate) practicing in 12 tertiary hospitals in 11 states across all six geopolitical zones participated in the study. None of the participating hospitals had a formal ASP programme and majority did not implement antimicrobial stewardship strategies. The median KAPPr scores were 71.1%, 77%, 75% and 53.3%, for the knowledge, attitude, practice, and prescription components, respectively. Only 22.3%, 40.3%, 31.6% and 31.7% of study respondents had good KAPPr, respectively. All respondents had prescribed one or more antibiotics in the prior 6 months, mostly Amoxicillin-clavulanate (98%), fluoroquinolones (97%), and ceftriaxone (96.8%). About 68% of respondents had prescribed antibiotics from the World Health Organization reserve group. Prior AMR training, professional rank, department, and hospital of practice were independently associated with good KAPPr.

**Conclusions:**

Our study suggests gaps in knowledge and attitude of APR and AMR with inappropriate prescriptions of antibiotics among physicians practicing in tertiary hospitals in Nigeria. Nigeria’s NAPAR should also target establishment and improvement of ASP in hospitals and address institutional, educational, and professional factors that may influence emergence of AMR in Nigeria.

## Introduction

The discovery of antibiotics has completely revolutionized medical practice and led to a decrease in the morbidity and mortality due to infectious diseases across the globe [1]. However, microbial evolution, misuse of antibiotics, and poor infection control practices, among other factors, have led to global emergence of antimicrobial resistance organisms (AMRO) with attendant difficult-to-treat infections, prolonged hospital stay, higher healthcare costs and poorer health outcomes [2, 3].

While the threat of antimicrobial resistance (AMR) remains a growing global challenge, developing countries in Asia and Africa are at greatest risk. It is estimated that by 2050, if nothing is done to halt the increasing trend of AMR, about 10 million people will die from AMR globally, including about 4 million people each from Asia and Africa [4]. At the 68th World Health Assembly, which held in May 2015, member countries endorsed a Global Action Plan (GAP) against AMR [5]. This plan required all countries to develop and implement a National Action Plan for Antimicrobial Resistance (NAPAR). The World Health Organization (WHO) is advocating that member countries adopt and implement the revised Model List of Essential Medicines which grouped antibiotics into the Access, Watch and Reserve (AWaRe) categories [6]. The Access group comprises essential antibiotics that should always be available. The Watch group consists of critically important antibiotics recommended only for specific limited indications, while the Reserve group are antibiotics that should be used as last resort when others have failed [6]. The AWaRe classification is intended to improve access to lifesaving antimicrobial medicines and prevent resistance due to excessive use of some priority antibiotics.

Infectious diseases remain the commonest cause of disease morbidity and mortality in Nigeria [7]. Following a country-wide situational analysis of antimicrobial use and resistance, Nigeria has identified AMR as an emerging health challenge deserving broad, good quality and locally relevant data to inform evidence-based interventions [8]. The focus areas of Nigeria’s 5-year (2017–2022) NAPAR are to: improve awareness and understanding of AMR and related topics; strengthen One Health AMR surveillance and research; improve infection prevention and control (IPC) in tripartite sector; promote rational access to antibiotics and antimicrobial stewardship; and invest in AMR-related research and development [9]. The country has since begun implementation of this plan through public and healthcare worker awareness creation and education, capacity building on laboratory surveillance and appropriate IPC. Nigeria enrolled in the Global Antimicrobial Resistance Surveillance System (GLASS) in April 2017 [10]. The country is currently expanding its laboratory capacity for antimicrobial susceptibility testing (AST): 21 laboratories have been enrolled into the National AMR surveillance system, while three laboratories submit routine reports on six priority bacterial pathogens to the GLASS [11].

Several studies within and outside Nigeria have shown that AMRO occur commonly among patients admitted in intensive care units, paediatric units and other specialist units often situated within tertiary hospitals [12, 13]. The major drivers of AMR in these settings are heavy antibiotic use and cross infection from hospital environment or personnel [14]. Tertiary hospitals are therefore potential breeding ground for the development and spread of AMRO in Nigeria.

Implementation of antimicrobial stewardship programmes (ASP) lies at the core of optimizing appropriate use of antibiotics in healthcare facilities [15, 16]. Any strategy to improve awareness and understanding of AMR and optimize the use of antibiotics among healthcare facilities in Nigeria, should evaluate implementation of ASP and ASM-related strategies among tertiary hospitals, and identify gaps in knowledge, attitude and practices (KAP) regarding antibiotic prescriptions (APR) and AMR among physicians.

Previous studies have shown that only 13–35% of tertiary hospitals in Nigeria had a formal ASP and majority did not implement most ASM-related strategies [17, 18]. Other studies conducted among physicians in tertiary hospitals in from revealed gaps in knowledge of AMR and poor practice of APR [19, 20]. However, none of these studies included participants from all six geopolitical zones of Nigeria and none assessed the predictors of appropriate KAP of APR and AMR among physicians in Nigeria.

We aimed to determine the KAP of APR and AMR, and identify sociodemographic, educational, institutional, and professional factors that may be associated with good KAP of APR and AMR among physicians in tertiary hospitals across all six geopolitical zones of Nigeria. Our overarching goal was to generate valuable sentinel national data to inform implementation of the country’s NAPAR.

## Methods

### Study design

This cross-sectional study was conducted between October 2018 and February 2019 among physicians practicing in public tertiary hospitals in Nigeria.

### Study population and setting

Nigeria runs a federal system of government consisting of a Federal Government, 36 semi-autonomous State Governments, the Federal Capital Territory (FCT) and 774 Local Government Areas. The states and FCT are grouped into six geo-political zones: South-South, South-East, South-West, North-East, North-West, and North Central. There are 101 public tertiary health facilities across all the 36 states and the FCT of Nigeria [21]. These public tertiary hospitals include federal and state-owned teaching hospitals, Federal Medical Centres and specialist hospitals. In Nigeria, tertiary hospitals provide both comprehensive and specialists healthcare to the general population; they are also centres for postgraduate medical training and health research.

### Study participants

We selected 12 tertiary hospitals across the country by convenience sampling and included at least one hospital from each of the six geopolitical zones of the country. In a bid to generate representative data, and allow for appropriate sub-group analysis, we targeted at least 40% of physicians working in each hospital. We then adopted a purposive sampling technique to guarantee inclusion of physicians of all professional ranks and departments in each of the selected hospitals. There were 4446 physicians in all 12 hospitals at the time of the study, and 1778 (40%) were eligible to be enrolled for the study.

### Data collection

A structured standardized self-administered pretested questionnaire was distributed to the study participants by trained research assistants who ensured that all completed questionnaires were returned the same day.

The questionnaire consisted of questions divided into four sections: (1) Implementation of antibiotic stewardship programmes (ASP) in selected hospitals and prior training on APR and AMR (2) Demographic and occupational characteristics of study participants; (3) Knowledge, attitude and practice of antibiotic prescriptions and resistance. (4) Frequency of prescriptions of 18 different antibiotics in the 6 months prior to the study.

The questionnaire was developed after reviewing qualitative and quantitative literatures for relevant items [22, 23], including prior surveys by the WHO [16, 24]. The questionnaire was further reviewed for face and content validity by experts in the field of ID and public health. This was then pre-tested in three states in a sample of 30 physicians who were not part of the study. Necessary adjustments were made before sending out the final updated questionnaires to respondents. The validity of the KAP questionnaire was confirmed by a Cronbach’s alpha internal consistency coefficient of ≥ 0.75 for each the 3 components.

### Implementation of antimicrobial stewardship programmes

A checklist consisting of 12 items assessed compliance with elements of ASP (see Fig. 2). The checklist was completed by appropriate staff as designated by the hospital management, including focal person on ASP or lead IPC team or Head Pharmacy unit, whichever is applicable.

A correct response to each ASP question was scored 1 while incorrect responses were scored 0. Thus, ASP scores range from 0 to 12.

### Knowledge, attitude, and practice

Participants’ knowledge of APR and AMR was assessed by a set of 28 questions covering general awareness about antibiotic use and resistance (eight questions), knowledge of rational use of antibiotics (eight questions), definition of AMR (one question) and knowledge of causes of antimicrobial resistance (11 questions). Correct responses were scored 1 while each wrong or do not know answer was scored 0. The overall knowledge score ranged from 0 to 28.

Attitude was assessed by a set of 15 positive and negative attitude questions using a five-point Likert’s scale. The scoring system used for positive attitude was: 5 = strongly agree with the statement, 4 = agree, 3 = neutral, 2 = disagree and 1 = strongly disagree with the statement. The scores were reversed for negative attitude questions. Overall attitude scores ranged from 15 to 75.

Practice was assessed by a set of 13 positive and negative practice questions using a five-point Likert’s scale. The scoring system used for correct practice was: 5 = Always, 4 = Most Often, 3 = Sometimes, 2 = Rarely and 1 = Never. The scores were reversed for wrong practice questions. Overall practice scores ranged from 13 to 65.

### Frequency of antibiotic prescriptions

To determine the frequency of prescriptions of 18 different antibiotics, respondents were asked to indicate how many times they prescribed each antibiotic in the previous 6 months using a Likert’s scale of Never, Rarely, Sometimes, Most Often, Always, scored 1, 2, 3, 4 and 5 respectively. Overall prescription scores ranged from 18 to 90.

### Classification of ASP, KAP and prescription scores

We calculated percentage scores for each ASP, KAP and prescription score. The 18 antibiotics were further classified into the WHO AWaRe categories [6]. At the time of this study, Cefepime was classified as a Reserve antibiotic. Prior prescriptions of the AWaRe categories of antibiotics were expressed in percentages.

Using modified Bloom’s cut-off point [25], the percentage ASP/KAP scores were grouped into good ASP/KAP (scores were between 80 and 100%), average ASP/KAP (scores between 50 and 79%) and poor ASP/KAP (scores less than 50%). The percentage prescription scores were also categorized into two groups: poor/very frequent prescriptions (prescription score between 50 and 100%) and good/less frequent prescription (prescription score less than 50%).

### Statistical analysis

The collected data were checked for completeness and errors before analysis. Incomplete questionnaires were excluded and counted as a non-response. All completed questionnaires were then entered into Microsoft Excel and exported to SPSS version 20 for analysis.

Study variables were summarized using frequencies, proportions, medians, and interquartile range-IQR (quantitative variables were not normally distributed). Spearman rho’s correlation coefficient was used to describe the strength and direction of the relationship among KAPPr scores. Correlations were interpreted using the following criteria: 0–0.25 = weak correlation, 0.25–0.5 = fair correlation, 0.5–0.75 = good correlation and greater than 0.75 = excellent correlation. A chi-squared test was used for bivariate analysis. For multivariate analysis, KAPPr scores were grouped into two: Good KAPPr (scores ≥ 80%) and Average/Poor KAPPr (scores < 80%). An unconditional binary logistic regression was used to determine independent variables associated with good KAPPr. Adjusted odds ratio with 95% confidence level were computed, and *p* value < 0.05 (two-sided) was considered statistically significant.

### Ethical consideration

All potential participants who agreed to participate provided written consent before completion of questionnaire. The questionnaire was anonymized, and confidentiality of information obtained was maintained. Ethical approval for the study was obtained from National Health Research Ethics Committee (NHREC/01/01/2017).

## Results

### Sociodemographic data

**Fig. 1:**
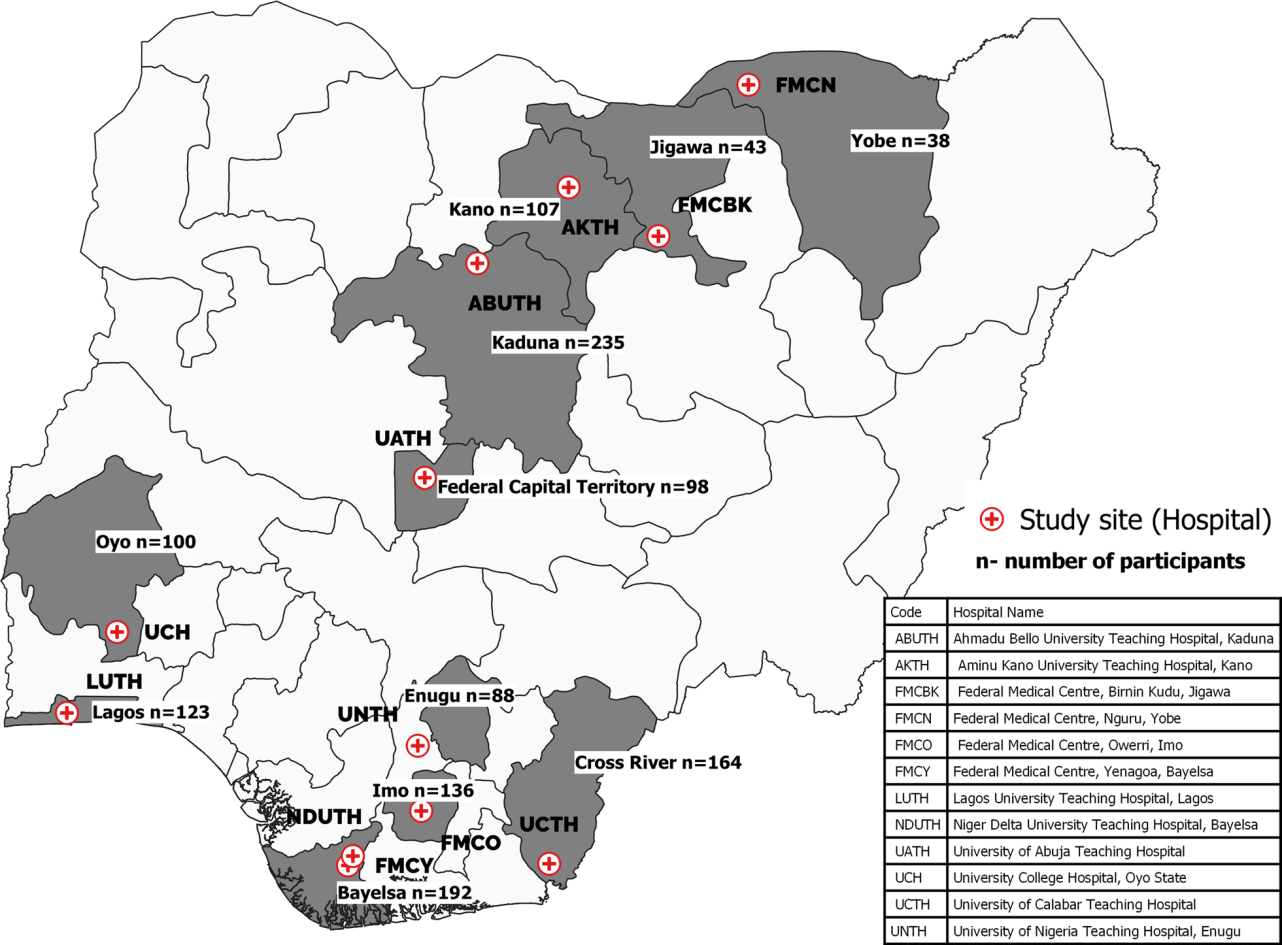
Geographical and hospital-based distribution of study participants

Of 1778 questionnaires, 1324 (74% response rate) were completed and returned from 11 states and 12 hospitals across all geopolitical zones of Nigeria (Fig. 1). Of the 1324 respondents, 889 (67.3%) were males, 1003 (75.6%) were between age 25 and 39 years and 834 (63%) were of the residency professional cadre. The south-south (26.9%) and the north-west (25.8%) regions had the highest proportions of respondents (Table 1).

**Table 1:** Demographic characteristics and professional features of respondents

Characteristics	Frequency (N = 1324)	Percent (%)
*Sex*		
Male	891	67.3
Female	433	32.7
*Age group*		
15–24 years	39	2.9
25–39 years	1003	75.8
40–59 years	264	19.9
Over 59 years	18	1.4
*Department*		
Emergency unit	35	2.7
Family medicine	81	6.1
Internal medicine	259	19.6
Obstetrics and gynaecology	514	38.8
Paediatrics	132	10.0
Public health	37	2.8
Surgery	163	12.3
Others	103	7.8
*Professional cadre*		
House officer	268	20.2
Medical officer	45	3.4
Resident	834	63.0
Consultant	177	13.4
*Prior training on AMR*		
Yes	417	31.5
No	907	68.5
*Region and Health Facility*		
North-central		
UATH	98	7.4
North-east		
FMCN	38	2.9
North-west		
ABUTH	235	17.7
AKTH	107	8.1
FMCBK	43	3.2
South-east		
UNTH	88	6.6
FMCO	136	10.3
South-south		
NDUTH	109	8.2
UCTH	164	12.4
FMCY	83	6.3
South-west		
UCH	100	7.6
LUTH	123	9.3

*UNTH* University of Nigeria Teaching Hospital, Enugu, *AKTH* Aminu Kano University Teaching Hospital, Kano, *NDUTH* Niger Delta University Teaching Hospital, Bayelsa, *UCH* University College Hospital, Oyo State, *LUTH* Lagos University Teaching Hospital, Lagos, *UCTH* University of Calabar Teaching Hospital, *UATH* University of Abuja Teaching Hospital

### Implementation of antimicrobial stewardship among hospitals and AMR training

**Fig. 2:**
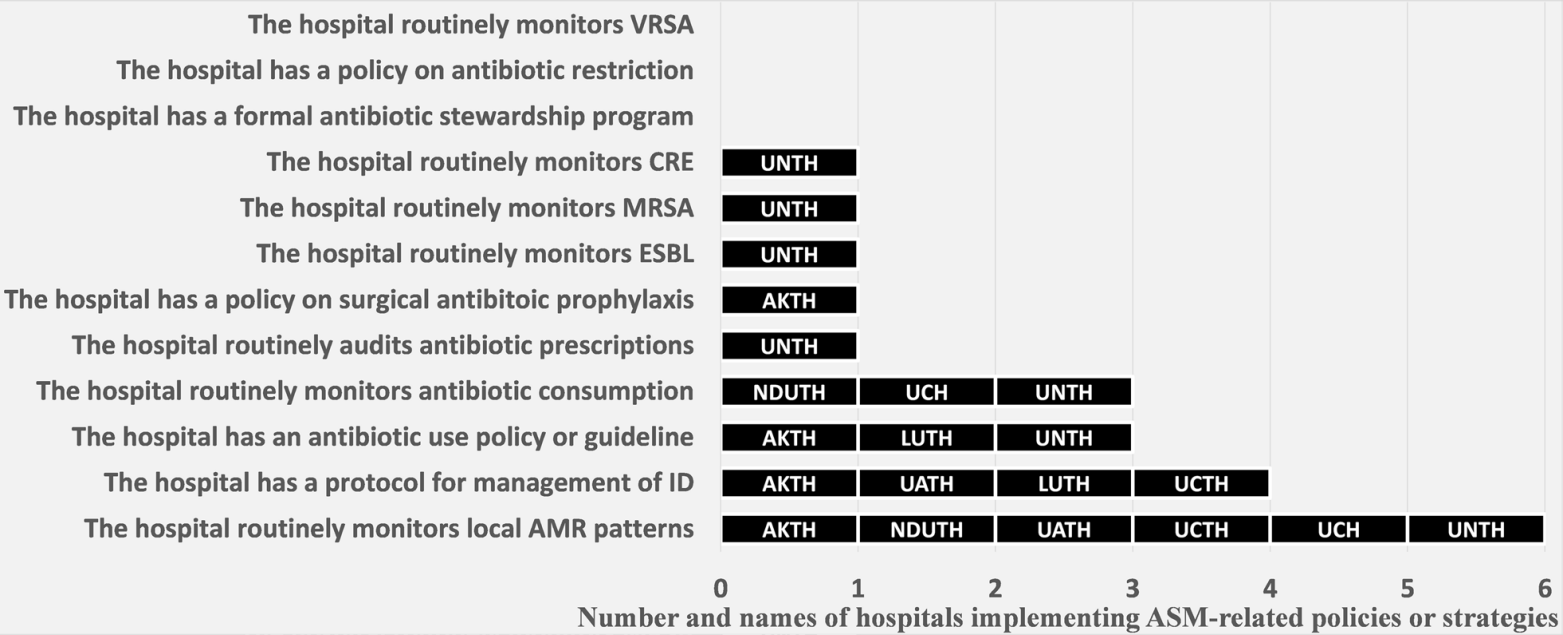
Implementation of antimicrobial stewardship programs and strategies by tertiary hospitals in Nigeria. None of the hospitals had a formal ASP or policy on antibiotic restriction. Half (50%) of the hospitals routinely monitored local AMR patterns. *UNTH* University of Nigeria Teaching Hospital, Enugu, *AKTH* Aminu Kano University Teaching Hospital, Kano, *NDUTH* Niger Delta University Teaching Hospital, Bayelsa, *UCH* University College Hospital, Oyo State, *LUTH* Lagos University Teaching Hospital, Lagos, *UCTH* University of Calabar Teaching Hospital, *UATH* University of Abuja Teaching Hospital

Out of a possible total score of 12, the ASP scores of hospitals surveyed ranged from 0 to 7, with median (and interquartile range-IQR) of 2 (0, 2). None of the hospitals surveyed had a formal ASP or a policy on antibiotic restrictions (Fig. 2). Three (25%) hospitals routinely monitored antibiotic consumption and had an antibiotic use policy, while four (33.3%) had a protocol for management of IDs in various departments. Although, 6 (50%) hospitals reported routine monitoring of antibiotic resistance patterns, only one (8.3%) routinely monitored specific AMRO such as ESBL, MRSA and CRE (Fig. 2). Only one hospital (UNTH) had above average ASP score (58.3%). All other hospitals had poor ASP score (between 0 and 33.3%).

Of the 1324 participants, 417 (31.5%) reported participating in a prior training on antibiotic use and resistance.

### Knowledge, attitude and practice of antibiotic use and resistance

The majority (> 80%) of study participants had heard of rational antibiotic prescription and AMR, but only 40.6% had heard of ASP (Table 2). Awareness of antibiotic resistant organisms ranged from 49 to 83%. Regarding knowledge of rational antibiotic use, 85.4% knew that antibiotics should only be stopped after completion of recommended doses, but 51% did not know that parenteral antibiotics are not necessarily more effective than oral antibiotics. Although 90% of participants knew that widespread use of antibiotics could promote emergence of AMR, most participants did not know that AMR could arise from use of antibiotics in farming and animal husbandry and from poor practice of hand hygiene in hospitals. The participants had lower knowledge scores for rational use of antibiotics compared to awareness about antibiotic use and resistance, and knowledge of causes of AMR (Table 2).

**Table 2:** Knowledge of antibiotic prescriptions and antimicrobial resistance among physicians in Nigeria

	Knowledge questions	% Correct
A	*Awareness of antibiotic use and resistance*	
	Have you previously heard about any of the following?	
1	Rational antibiotic use	89.6
2	Antibiotic resistance	97.7
3	Antibiotic stewardship program	40.6
4	Antibiogram	45.3
5	Methicillin-resistant *Staphylococcus aureus* (MRSA)	83.2
6	Vancomycin-resistant *Staphylococcus aureus* (VRSA)	74.2
7	Extended spectrum beta-lactamases (ESBL)	65.3
8	Carbapenem-resistant Enterobacteriaceae (CRE)	48.9
	Overall % correct—median (IQR) = 75 (62.5, 87.5)	
B	*Knowledge of rational antibiotic use*	
	Concerning antibiotic use which of the following statements are correct?	
9	Antibiotics should not be used to treat non-bacterial infections	76.6
10	Antibiotics may be used to treat common cold	71.5
11	Antibiotics should be avoided in cases of acute diarrhoea	51.5
12	Antibiotics should be stopped as soon as patient symptoms resolve	84.1
13	Antibiotics should only be stopped after completing recommended doses	85.4
14	Parenteral antibiotics are more effective than oral antibiotics	49
15	Prophylactic surgical antibiotic should be discontinued after 24hours	39.4
16	Prophylactic surgical antibiotic should not be given for less than 3 days	47.1
	Overall % correct—median (IQR) = 62.5 (50, 75)	
C	*Knowledge of definition of AMR*	
17	Antibiotic resistance means the micro-organism is resistant to the antibiotic	69.3
D	*Knowledge of causes of AMR*	
	The following are known to promote emergence of antibiotic resistance?	
18	Widespread use of antibiotics	90
19	Use of broad-spectrum antibiotics	56.6
20	Antibiotic use in animal husbandry	41.5
21	Antibiotic use in farming	39.7
22	Poor practice of hand hygiene in hospitals	47.7
23	Prescribing parenteral antibiotics	62.6
24	Lack of antibiotics prescribing guidelines	91.8
25	Microbial mutations	94.3
26	Premature interruption of antibiotics	92.7
27	Use of antibiotics to treat common cold	74.5
28	Vaccination	74.5
	Overall % correct—median (IQR) = 72.7 (63.6, 81.8)	

Concerning attitude toward antibiotic use and resistance, majority of participants agreed that AMR is a serious public health issue in Nigeria and worldwide and could be a problem for their hospital (Table 3). However, about 79% disagreed that routine hand washing could prevent AMR, 50% agreed that pharmaceutical companies could influence their choice of antibiotic prescriptions, and 27.9% wrongly believed that antibiotic could be used to prevent bacterial infection in patients with upper respiratory tract infection (URTI) due to viruses.

**Table 3:** Attitude and practice of antibiotic prescriptions and antimicrobial resistance among physicians in Nigeria

Sn	Attitude	Strongly agree	Agree	Neutral	Disagree	Strongly disagree
		%	%	%	%	%
1	Antibiotic resistance is a serious public health issue worldwide	79.3	18.5	1.8	0.3	0.2
2	Antibiotic resistance is a serious public health issue in Nigeria	82.0	16.3	1.1	0.2	0.4
3	Antibiotic resistance is a problem in other hospitals but not in our hospital	1.3	2.2	6.4	46.8	43.3
4	Antibiotic could be used to prevent bacterial infection in patients with viral URTI	6.6	20.4	12.3	29.6	31.2
5	Any patient with fever would benefit from antibiotic therapy	1.8	6.3	9.1	43.8	38.9
6	Prolonged use of broad-spectrum antibiotics is a risk factor for antibiotic resistance	42.9	38.9	7.0	7.4	3.8
7	Patients may feel better if you prescribe antibiotics to satisfy their demands and expectations	4.3	22.3	17.9	29.8	25.7
8	There is nothing I can do as a person to lower the risk of antibiotic resistance in our hospital	2.2	1.9	6.1	38.6	51.2
9	There is no risk of resistance if antibiotics are taken as prescribed	21.1	32.4	20.4	17.6	8.5
10	Persons who have never taken antibiotics have no risk of resistance	8.0	19.1	10.1	46.1	16.8
11	It is better to stop antibiotics as soon as a patient feels better	3.5	11.3	11.5	49.7	24.0
12	Regular hand washing can prevent antibiotics resistance	4.6	7.9	8.3	42.6	36.6
13	A longer course of antibiotic is less likely to cause resistance than a short course	7.8	19.2	18.6	37.7	16.7
14	Pharmaceutical companies sometimes influence my choice of antibiotics	9.1	40.5	15.2	24.0	11.2
15	In hospital setting, antibiotic resistance could be transmitted from healthcare worker to patients	26.7	37.2	12.0	15.2	8.9

	Practice	Always	Most of the time	Sometimes	Rarely	Never
		%	%	%	%	%
1	Prescribe antibiotics for common cold	1.4	3.3	28.0	44.1	23.2
2	Prescribe antibiotics for pneumonia	45.4	45.1	6.3	1.5	1.7
3	Prescribe antibiotics for malaria	2.0	1.5	8.1	21.7	66.7
4	Stop antibiotics immediately patient symptoms resolve	1.4	5.0	15.8	26.5	51.4
5	Prescribe antibiotics because patient insists on it	0.2	0.8	11.2	27.8	60.0
6	Prescribe antibiotics because you do not trust the available laboratory results	1.1	8.6	61.9	17.4	10.9
7	Wait for culture result before prescribing antibiotics	0.8	10.6	49.2	30.5	8.9
8	Prescribe antibiotics based on culture results	17.9	48.0	28.7	3.8	1.6
9	Prescribe antibiotic based on recommendation of pharmaceutical companies	1.4	4.8	29.5	37.7	26.6
10	Prescribe antibiotics inappropriately because patient cannot afford the appropriate antibiotic	0.4	3.4	34.0	29.0	33.2
11	Prescribe antibiotics inappropriately because the appropriate antibiotic is not available	0.6	3.4	38.1	30.2	27.8
12	De-escalate from broad spectrum to narrow spectrum antibiotics as soon as culture results are available	24.0	37.1	22.3	9.9	6.7
13	Prescribe prophylaxis antibiotics for more than 24hours	6.4	19.2	41.9	23.3	9.1

*URTI* upper respiratory tract infection

Table 3 shows that about 62% of participants sometimes prescribe antibiotics because they did not trust the available laboratory results, 43% have ever prescribed antibiotics for malaria and 28% sometimes prescribe antibiotics for common cold. About 78% of participants stated that they had rarely or never stopped antibiotics immediately patient symptoms resolve, and 29.5% had sometimes prescribe antibiotics based on recommendation of pharmaceutical companies.

### Antibiotic prescriptions

**Fig. 3:**
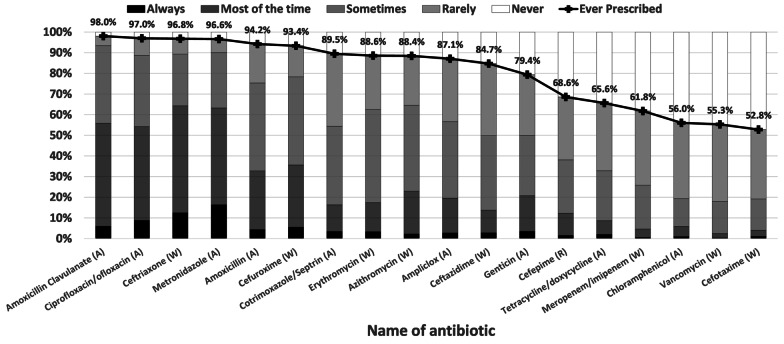
Frequency of prescription of various antibiotics by physicians in Nigeria in the prior 6 months (based on Likert’s scale). Penicillins with β-lactamase inhibitors, fluoroquinolones and third generation cephalosporins were the most frequently classes of antibiotics prescribed. 68.6% had prescribed the reserved antibiotic Cefepime

All study participants (100%) had prescribed one or more antibiotics in the previous 6 months. A median prescription of 15 antibiotics (IQR-13, 17) were prescribed per study participant in the previous 6 months. The prescription scores ranged from 20 to 90 with median (IQR) of 48 (43, 53). Amoxicillin-clavulanate (98%), ciprofloxacin/ofloxacin (97%), ceftriaxone (96.8%) and metronidazole (96.5%) were the most frequently prescribed antibiotics (Fig. 3). According to AWaRe categories, 100%, 99.3%, 67.8% of respondents had prescribed antibiotics from the Access, Watch and Reserve group of antibiotics, respectively.

**Fig. 4:**
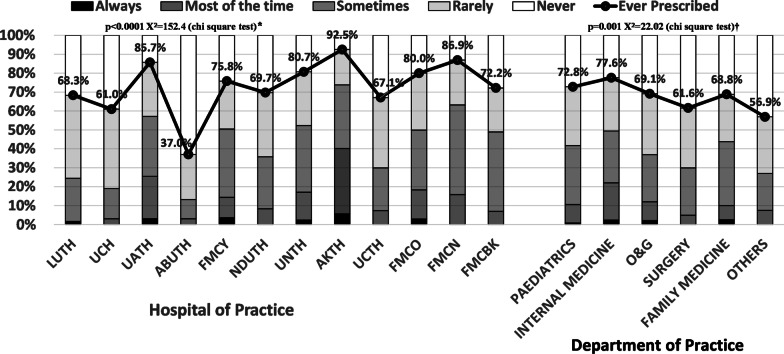
Frequency of prescription of reserve antibiotic (Cefepime) among physicians in Nigeria. There was significant difference in prescription of Cefepime in relation to hospital of practice* and department of practice†. Physicians practicing in AKTH (92.5%), FMCN (85.9%) and UATH (85.7%), all located in northern Nigeria, had the highest rates prescriptions of Cefepime. The highest rates of prescription of Cefepime were also observed among physicians practicing in and Internal Medicine (77.6%) and Paediatrics (72.8%) departments. *UNTH* University of Nigeria Teaching Hospital, Enugu, *AKTH* Aminu Kano University Teaching Hospital, Kano, *NDUTH* Niger Delta University Teaching Hospital, Bayelsa, *UCH* University College Hospital, Oyo State, *LUTH* Lagos University Teaching Hospital, Lagos, *UCTH* University of Calabar Teaching Hospital, *UATH* University of Abuja Teaching Hospital, *FMCN* Federal Medical Centre, Nguru, Yobe, *FMCY* Federal Medical Centre, Yenagoa, Bayelsa, *FMCO* Federal Medical Centre, Owerri, Imo, *FMCBK* Federal Medical Centre, Birnin Kudu, Jigawa, *ABUTH* Ahmadu Bello University Teaching Hospital, Kaduna

Respondents practicing in FMC Nguru, Yobe State and AKTH, Kano and those working in Paediatrics and Internal Medicine departments reported higher rates of prescriptions of Cefepime than respondents from other hospitals and departments (Fig. 4).

### Correlations between knowledge, attitude, practice, and prescription (KAPPr) scores

The correlations between KAPPr scores are shown in Table 4. There were weak positive correlations between knowledge, attitude, and practice scores, and weak negative correlations when prescriptions scores are compared with knowledge, attitude, and practice scores.

**Table 4:** Spearman rho’s correlations between knowledge, attitude, practice, and prescription scores

Scores	Knowledge score-r (*p* value)	Attitude score-r (*p* value)	Practice score-r (*p* value)	Prescription score-r (*p* value)
Knowledge score	1	0.360 (< 0.0001)	0.191 (< 0.0001)	− 0.074 (0.008)
Attitude score	0.360 (< 0.0001)	1	0.296 (< 0.0001)	− 0.217 (< 0.0001)
Practice score	0.191 (> 0.0001)	0.296 (< 0.0001)	1	− 0.192 (< 0.0001)
Prescription score	− 0.074 (0.008)	− 0.217 (< 0.0001)	− 0.192 (< 0.0001)	1

NB: there were weak positive correlations between knowledge, attitude and practice scores and weak negative correlations between when prescription scores where compared with knowledge, attitude, and practice scores

### Prevalence and factors associated with good knowledge, attitude, practice, and prescription.

The descriptive statistics of KAPPr scores are summarized in Table 5. Of the 1324 study participants, 295 (22.3%), 534 (40.3%), 418 (31.6%) and 420 (31.7%) had good knowledge, attitude, practice, and prescription, respectively. The factors associated with good KAPPr on univariate and multivariate analysis are shown in Tables 6 and 7, respectively. Professional rank was independently associated with good knowledge, attitude, and prescription but not with good practice. Senior physicians (consultants and resident doctors) were between 1.6 and 3.3 times more likely to have good KAPPr than house officers. Those who previously participated in any training on antibiotic use and resistance were 2.6 times more likely to have good knowledge than those without any prior training.

**Table 5:** Descriptive statistics of knowledge, attitude, practice, and prescription scores of physicians in Nigeria

Variable	Score	Score (n)	Score (%)	Good score	Average score	Poor score
	Range	Median (IQR)	Median (IQR)	n (%)	n (%)	n (%)
Knowledge	0–28	19 (16, 21)	71.1(62, 79)	293 (22.3)	963 (73.4)	56 (4.3)
Attitude	15–75	57 (52, 62)	77 (72, 83)	531 (40.3)	786 (59.6)	1 (0.1)
Practice	13–65	49 (46, 52)	75 (72, 80)	416 (31.6)	899 (68.4)	0 (0)
Prescription	18–90	48 (43, 53)	53.3 (47.8, 58.9)	420 (31.7)	896 (67.7)	8 (0.6)

*n* number of participants, *IQR* interquartile range

**Table 6:** Univariate analysis of factors associated with good knowledge, attitude, practice, and prescription among physicians in Nigeria

Variable	N	Good knowledge	Good attitude	Good practice	Good prescription
		n (%)	n (%)	n (%)	n (%)
*Gender*					
Female	430	84 (19.8)	177 (41.5)	138 (32.5)	149 (34.7)
Male	889	208 (23.6)	353 (39.8)	277 (31.3)	268 (30.1)
*Age group*					
< 36 years	817	146 (18.1)*	298 (36.7)*	238 (29.4)*	244 (29.9)
36–49 years	456	128 (28.1)	206 (45.3)	151 (33.3)	160 (35.1)
> 49 years	51	19 (37.3)	27 (52.9)	27 (52.9)	16 (31.4)
*Professional rank*					
House officers	268	33 (12.5)*	71 (26.9)*	69 (26.2)*	61 (22.8)
Medical officers/residents	879	197 (22.6)	362 (41.2)	290 (33.1)	280 (31.9)
Consultants	177	63 (35.6)	98 (55.7)	57 (32.4)	79 (44.6)
*Prior AMR training*					
No	870	142 (16.5)*	323 (37.3)*	257 (29.8)*	279 (32.1)
Yes	417	143 (34.5)	190 (45.7)	147 (35.3)	132 (31.7)
*Department*					
Paediatrics	132	30 (22.9)*	54 (41.2)	38 (29)*	23 (17.4)*
Internal medicine	259	75 (29)	121 (46.9)	81 (31.5)	77 (29.7)
O&G	515	105 (20.5)	197 (38.5)	150 (29.5)	161 (31.3)
Surgery	164	42 (25.8)	67 (41.1)	58 (35.4)	60 (36.6)
Family medicine	80	14 (18.2)	25 (31.2)	39 (48.8)	22 (27.5)
Others	174	27 (15.9)	67 (38.5)	50 (28.7)	77 (44.3)
*Hospital*					
LUTH	123	50 (40.7)*	86 (69.9)*	52 (42.3)*	45 (36.6)*
UCH	100	37 (37.4)	51 (52)	40 (40.8)	47 (47)
UATH	98	25 (25.5)	41 (42.3)	37 (37.8)	24 (24.5)
ABUTH	235	48 (20.4)	103 (43.8)	63 (26.9)	80 (34)
FMCY	83	13 (16.2)	18 (21.7)	33 (39.8)	24 (28.9)
NDUTH	109	21 (19.4)	32 (29.6)	39 (36.1)	34 (31.2)
UNTH	88	12 (13.6)	23 (26.1)	13 (15.1)	25 (28.4)
AKTH	107	29 (28.2)	45 (42.1)	19 (17.8)	30 (28)
UCTH	164	27 (16.5)	52 (31.7)	61 (37.2)	57 (34.8)
FMCO	136	27 (20.3)	62 (46.3)	42 (31.6)	39 (28.7)
FMCN	38	2 (5.3)	7 (18.4)	9 (23.7)	5 (13.2)
FMCBK	43	2 (4.7)	11 (25.6)	8 (18.6)	10 (23.3)
*All participants*	1324	293 (22.3)	531 (40.3)	416 (31.6)	420 (31.7)

*N* number of participants, *AMR* antimicrobial resistance, *UNTH* University of Nigeria Teaching Hospital, Enugu, *AKTH* Aminu Kano University Teaching Hospital, Kano, *NDUTH* Niger Delta University Teaching Hospital, Bayelsa, *UCH* University College Hospital, Oyo State, *LUTH* Lagos University Teaching Hospital, Lagos, *UCTH* University of Calabar Teaching Hospital, *UATH* University of Abuja Teaching Hospital, *FMCN* Federal Medical Centre, Nguru, Yobe, *FMCY* Federal Medical Centre, Yenagoa, Bayelsa, *FMCO* Federal Medical Centre, Owerri, Imo, *FMCBK* Federal Medical Centre, Birnin Kudu, Jigawa, *ABUTH* Ahmadu Bello University Teaching Hospital, Kaduna

*p < 0.05

**Table 7:** Multivariate analysis of the predictors of good knowledge, attitude, practice, and prescription among physicians in Nigeria

Variable	Good knowledge	Good attitude	Good practice	Good prescription
	AOR (95% CI)	AOR (95% CI)	AOR (95% CI)	AOR (95% CI)
*Gender*				
Female (Ref)	1	1	1	1
Male	1.5 (1, 2.1)	1.1 (0.9, 1.5)	1 (0.8, 1.4)	0.8 (0.6, 1)
*Age group*				
< 36 years (Ref)	1	1	1	1
36–49 years	1.2 (0.9, 1.7)	1 (0.7, 1.3)	1.1 (0.8, 1.5)	1 (0.8, 1.3)
> 49 years	1.7 (0.8, 3.5)	1.2 (0.6, 2.4)	2.7^*^ (1.4, 5.4)	0.6 (0.3, 1.1)
*Professional category*				
House officers	1*	1*	1	1*
Medical officers/residents	1.6* (1, 2.5)	1.8^*^ (1.2, 2.5)	1.4 (1, 2)	1.6* (1.1, 2.3)
Consultants	2.4* (1.3, 4.5)	3^*^ (1.8, 5)	1 (0.6, 1.7)	3.3^*^ (2, 5.7)
*Prior AMR training*				
No (Ref)	1*	1	1	1
Yes	2.6 (1.9, 3.4)	1.3 (1, 1.7)	1.2 (0.9, 1.5)	0.9 (0.7, 1.2)
*Department*				
Paediatrics (Ref)	1	1	1	1*
Internal medicine	1.3 (0.7, 2.2)	1.2 (0.7, 2)	1.3 (0.8, 2.1)	2.2^*^ (1.3, 3.9)
O&G	1.1 (0.6, 1.9)	0.8 (0.5, 1.3)	1.2 (0.7, 1.9)	2.7^*^ (1.6, 4.6)
Surgery	0.9 (0.5, 1.6)	0.8 (0.5, 1.4)	1.3 (0.8, 2.2)	2.9^*^ (1.6, 5.2)
Family medicine	0.7 (0.3, 1.5)	0.6 (0.3, 1.1)	2.3 (1.2, 4.2)	1.9 (0.9, 3.8)
Others	0.6 (0.3, 1.2)	0.8 (0.5, 1.3)	1.1 (0.7, 1.9)	4.5^*^ (2.5, 8)
*Hospital*				
LUTH (Ref)	1*	1*	1*	1*
UCH	0.9 (0.5, 1.6)	0.5* (0.3, 0.9)	0.8 (0.5, 1.4)	1.8* (1, 3.2)
UATH	0.5 (0.3, 1)	0.4* (0.2, 0.7)	0.8 (0.4, 1.4)	0.7 (0.4, 1.4)
ABUTH	0.4* (0.2, 0.7)	0.3* (0.2, 0.6)	0.5* (0.3, 0.8)	0.8 (0.5, 1.3)
FMCY	0.3* (0.1, 0.6)	0.1*(0.1, 0.2)	0.7 (0.4, 1.4)	0.9 (0.5, 1.8)
NDUTH	0.3* (0.2, 0.6)	0.2* (0.1, 0.4)	0.7 (0.4, 1.3)	1 (0.6, 1.9)
UNTH	0.2* (0.1, 0.5)	0.2* (0.1, 0.4)	0.3* (0.1, 0.6)	0.7 (0.4, 1.4)
AKTH	0.6 (0.3, 1.1)	0.3* (0.2, 0.5)	0.3* (0.2, 0.6)	0.6 (0.3, 1.1)
UCTH	0.3* (0.2, 0.6)	0.2* (0.1, 0.4)	0.8 (0.5, 1.4)	1.2 (0.7, 2)
FMCO	0.3* (0.2, 0.7)	0.5* (0.3, 0.9)	0.7 (0.4, 1.2)	0.8 (0.4, 1.4)
FMCN	0.1* (0, 0.4)	0.1* (0, 0.3)	0.4 (0.2, 1)	0.3 (0.1, 1)
FMCBK	0.1* (0, 0.3)	0.1* (0.1, 0.3)	0.3* (0.1, 0.7)	0.5 (0.2, 1.2)

*Ref* reference group, *AOR* adjusted odds ratio, *CI* confidence interval, *AMR *antimicrobial resistance, *UNTH* University of Nigeria Teaching Hospital, Enugu, *AKTH* Aminu Kano University Teaching Hospital, Kano, *NDUTH* Niger Delta University Teaching Hospital, Bayelsa, *UCH* University College Hospital, Oyo State, *LUTH* Lagos University Teaching Hospital, Lagos, *UCTH* University of Calabar Teaching Hospital, *UATH* University of Abuja Teaching Hospital, *FMCN* Federal Medical Centre, Nguru, Yobe, *FMCY* Federal Medical Centre, Yenagoa, Bayelsa, *FMCO* Federal Medical Centre, Owerri, Imo, *FMCBK* Federal Medical Centre, Birnin Kudu, Jigawa, *ABUTH* Ahmadu Bello University Teaching Hospital, Kaduna

*p < 0.05

**Fig. 5:**
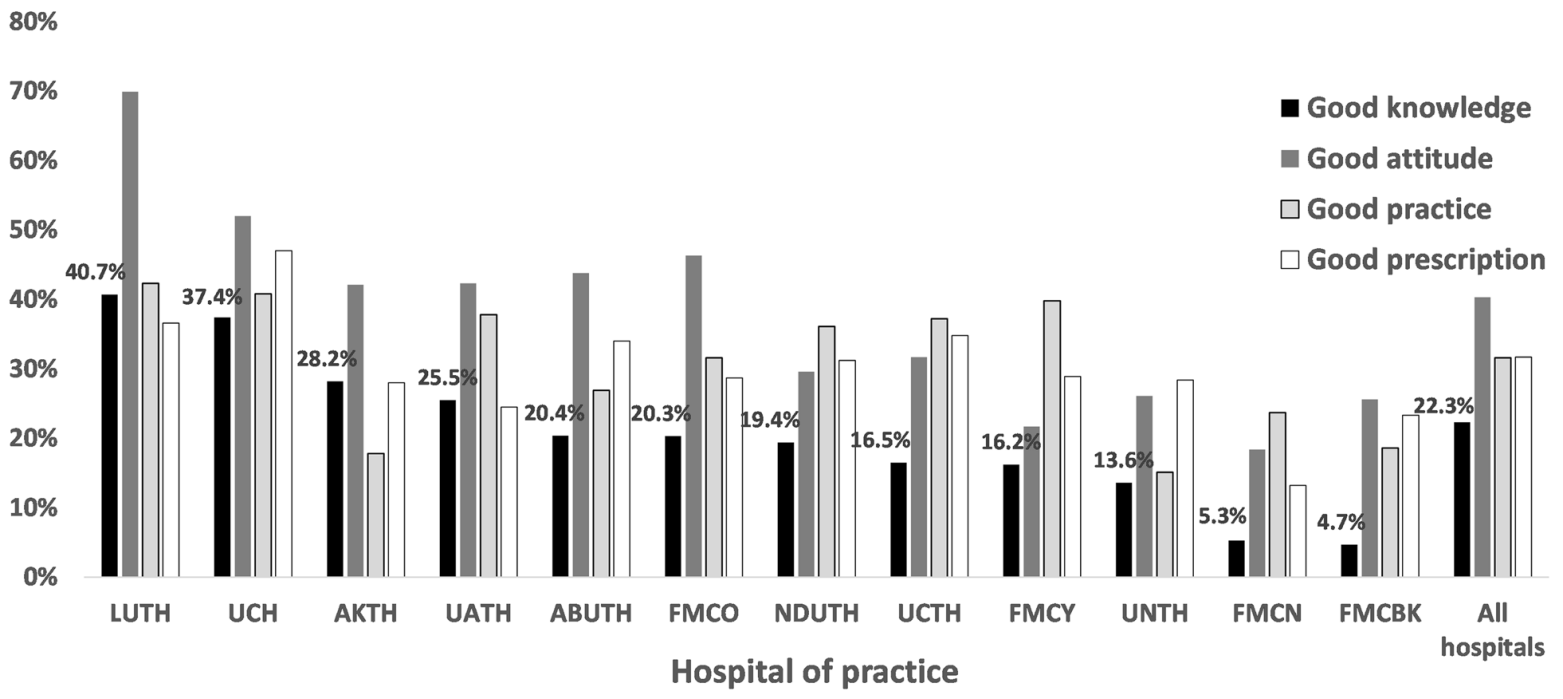
Differences in prevalence of good knowledge, attitude, practice, and prescription in relation to hospital of practice of physicians in Nigeria. LUTH and UCH both located in South-West Nigeria generally had higher prevalence of good KAPPr than other hospitals. *UNTH* University of Nigeria Teaching Hospital, Enugu, *AKTH* Aminu Kano University Teaching Hospital, Kano, *NDUTH *Niger Delta University Teaching Hospital, Bayelsa, *UCH* University College Hospital, Oyo State, *LUTH* Lagos University Teaching Hospital, Lagos, *UCTH* University of Calabar Teaching Hospital, *UATH* University of Abuja Teaching Hospital, *FMCN* Federal Medical Centre, Nguru, Yobe, *FMCY* Federal Medical Centre, Yenagoa, Bayelsa, *FMCO* Federal Medical Centre, Owerri, Imo, *FMCBK* Federal Medical Centre, Birnin Kudu, Jigawa, *ABUTH* Ahmadu Bello University Teaching Hospital, Kaduna

Department of practice was independently associated with good prescription, but not associated with good knowledge, attitude, and practice. Respondents practicing in paediatrics departments were significantly less likely to have good prescription compared to respondents from other departments. Hospital of practice was independently associated with good KAPPr. Overall, respondents practicing in South West Hospitals (UCH and LUTH) were more likely to have good KAPPr than those respondents from other hospitals (Fig. 5). Respondents practicing in FMCN and FMCBK, both located in northern Nigeria, generally had lowest rates of good KAPPr.

## Discussion

To the best of our knowledge, this is the first study from Nigeria assessing both implementation of ASM among tertiary hospitals and predictors of good KAP of APR and AMR among physicians practicing in all six geopolitical zones of Nigeria.

Our results suggest significant gaps in the implementation of antimicrobial stewardship (AMS). None of the hospitals surveyed had a formal ASP program, while majority of participating hospitals were not routinely implementing one or more of the ASM-related interventions and there was poor awareness of ASM. Poor compliance with AMS was reported by another study from Nigeria where only six (35%) of 17 tertiary hospitals surveyed had a formal ASP and less than 23.5% of these hospitals implemented AMS-related interventions and policies [17]. A nationwide survey of pharmacist’s involvement in ASP in Nigeria, revealed that only 5 (13.5%) of 37 hospitals had a formal ASP. In a study evaluating physicians’ knowledge and perception of AMS and AMR in six hospitals in three geopolitical zones of Nigeria, only 28.2% of respondents had ever heard of ASP and more respondents had good knowledge of AMR than ASP (82.7% vs 36.5%) [19]. In line with our finding of poor implementation of AMS-related interventions, an international survey of ASP in 660 hospitals across the globe, revealed that only 14% of 43 hospitals in Africa had an ASP in place as compared to 53% (26/49), 66% (230/348) and 66% (45/67) of hospitals in Asia, Europe, and North America, respectively [26]. This international survey reported lack of funding and personnel, a lack of information technology, prescriber opposition and lack of awareness on the part of the hospital administration as perceived barriers to the implementation of ASP among countries surveyed. Other identified challenges to implementation of ASP in developing countries include, limited diagnostic infrastructure, poor access to quality-assured antimicrobials, and gaps in antibiotic prescribing and AMR-related knowledge and attitude among prescribers [27, 28]. Although, the outcomes and impact of implementation of ASP have not been widely studied in Africa, few studies from this region suggest that African countries can successfully implement these programmes [29]. To overcome these barriers and challenges, Nigeria ought to strengthen its antimicrobial stewardship through advocacy for institutional buy-in, training and education and appropriate funding of AMS activities.

Our study respondents had better knowledge of AMR (median score of 72.7%) than knowledge of rational APR (median score of 62.5%). Most respondents agreed that AMR is a serious problem in their various hospitals and Nigeria as a whole. However, over half of our respondents did not know that AMR could arise from antibiotic use in animal husbandry and farming. About 78% of farms in Nigeria are using antimicrobials for animal production, and 20% of these livestock have been shown to have multi-drug resistant isolates [30]. Our results support the need to improve awareness on the association between antimicrobial use in animals and emergence of AMR in humans, and to institutionalize national antimicrobial use and resistance surveillance system in animals in Nigeria.

The gap in knowledge of the link between poor practice of IPC and AMR as observed in our study may partly explain why 79% of respondents disagreed that routine hand washing could prevent AMR and 36% were neutral or disagreed that AMR could be transmitted from healthcare worker to patient. About 83% of physicians in another study from Nigeria identified poor infection control as a possible cause of emergence of AMR in hospital settings [19]. However, this study did not specifically evaluate hand hygiene-related knowledge and attitude. Garba et al. reported that 54.1% of healthcare workers in primary healthcare centers in Kaduna North local government area of northern Nigeria wrongly believed that practice of hand hygiene for prevention of AMR is overrated [31]. A study from Europe reported that junior doctors rarely perceived poor hand hygiene practices as important drivers of AMR [32]. The slogan 'Fight antibiotic resistance—it's in your hands' was formulated in 2017 by the WHO to emphasize the central role of hand hygiene in the prevention of AMR [33]. Our study findings suggest that there is need to improve awareness among physicians in Nigeria about the importance of practice of hand hygiene and other components of IPC in reducing the transmission of AMRO in hospital settings.

The median KAP scores of respondents in our study were 71%, 75% and 77% respectively. However, only 22.3%, 40.3%, and 31.6 had good KAP (defined as score of 80% or above) of APR and AMR, respectively. In a systematic review of KAP of APR and AMR among healthcare practitioners from developing countries involving 15 studies (none from Nigeria) an average of 80.9% of respondents correctly answered questions relating to APR, whereas only 39.6% were aware of the local resistance patterns in their health facilities [34]. About 83% of physicians working in tertiary hospitals in four geopolitical zones of Nigeria were reported to have good knowledge of AMR (defined as knowledge score of 80% or above) [19]. Among healthcare practitioners from primary health facilities in northern Nigeria, 73% had good knowledge of AMR (defined as knowledge score between 50 and 74%), while less than 5% had very good knowledge of AMR (defined as knowledge score of 75% and above) [31]. The variability in the proportions of respondents with good knowledge from Nigeria could be related to differences in study design. It is our view that our study findings are representative of physicians working in tertiary hospitals in Nigeria as we included hospitals and physicians across all six geopolitical zones of the country.

Excessive and inappropriate prescription of antibiotics are established drivers of AMR [35, 36]. Our results reveal that an average of 15 (83.3%) of 18 antibiotics were prescribed by each respondent in the prior 6 months. About 69% of respondents were classified as having poor prescription practice, meaning they prescribed antibiotics inappropriately. Some indicators of inappropriate APR in our study included 43% of respondents ever prescribing antibiotics for malaria and 28% sometimes prescribing antibiotics for common cold. Our results also reveal some attitudes and drivers of irrational APR including influence of pharmaceutical companies, lack of trust in laboratory test results, attempting to satisfy patient expectations, prohibitive costs of some antibiotics, and limited access to appropriate antibiotic. Penicillins with β-lactamase inhibitors, fluoroquinolones and third generation cephalosporins were the most common classes of antibiotics frequently prescribed by respondents in our study. While most respondents had prescribed antibiotics from the WHO Access and Watch categories, it is remarkable that about 63% of respondents had prescribed antibiotics in the Reserve group of the WHO AWaRe categories. The high rates of prescription of the Reserve antibiotics Cefepime without recourse to culture results could ultimately lead to high burden of ESBL.

Many studies from Nigeria have also reported overuse and irrational prescription of antibiotics among physicians, with prevalence of self-reported APR ranging from 26.8 to 97% [19, 20, 37] and point prevalence surveys (PPS) reporting APR rates of 59.6–80.1% among in-patients [38–41]. The global PPS reported a higher prevalence of antibiotic prescribing among Africa hospitals (average of 50%) compared to 27.4% among European hospitals [13]. Most of these studies also reported Penicillins with β-lactamase inhibitors, third generation cephalosporins and fluoroquinolones as the commonest classes of antibiotics prescribed.

The determinants of inappropriate prescriptions of antibiotics have been reviewed by various authors [42, 43]. A systematic review of physicians practicing in different settings across the globe identified patients’ expectations, severity and duration of infections, uncertainty over diagnosis, potentially losing patients, and influence of pharmaceutical companies as drivers of irrational APR among physicians [22]. A study of physicians from a tertiary hospital in Lagos, Nigeria reported factors of cost, drug availability and pressure from pharmaceutical representatives as the major drivers of irrational APR among study participants [20].

Educational programmes have been shown to improve awareness and knowledge of AMR among healthcare workers, as well as foster appropriate prescription behaviour [44, 45]. We found that good knowledge of AMR and APR was associated with prior training on antibiotic use and resistance. However, the lack of association between prior training and good attitude, practice, and prescription, as well as the weak positive correlations between KAP, support the role of multiplicity of factors in determining attitudes and practices regarding APR and AMR [37, 46, 47]. Additional predictive factors observed in our study were professional rank, hospital and department of practice of physicians.

Independent of other variables, more senior physicians (consultants and resident doctors) in our study had better knowledge and attitudes, and prescribed antibiotics less frequently than recently qualified medical interns. Medical interns have limited experience of clinical practice, and it is not surprising they had the lowest KAPPr scores in our study. Besides poor knowledge, the more frequent prescriptions of antibiotics among these interns compared to other physicians could be related to the hierarchical nature of medical practice in Nigeria where medical interns are in most cases required to write out prescriptions based on instructions of their supervising residents or consultants. The rotation of interns through all major medical specialties has been shown to influence their prescription practice as they are made to transcribe prescriptions from a wide variety of their supervisors in the various departments [48]. Studies from Ghana [49], USA [50], Malaysia [51] and China [44] have also reported higher knowledge and less frequent prescriptions of antibiotic among senior physicians compared to junior physicians. Other studies have shown that prescription errors are more frequent among medical interns in hospital settings [52, 53].

Hospital of practice was independently associated with good KAPPr among physicians in our study. Physicians practicing in hospitals located in the South-West region of the country generally had better KAPPr scores than those from other hospitals. Prescriptions of Reserve antibiotic (Cefepime) were more frequently observed among physicians practicing in northern Nigeria, as all three hospitals with over 80% of physicians with prior prescriptions of Cefepime were in northern Nigeria. Among five published PPS from Nigeria [38–41, 54], the lowest prevalence of antibiotic use among inpatients of 59.5% was reported in a South-West hospital [41] while the highest prevalence of 80.1% was reported among three hospitals located in northern Nigeria [39]. The reasons for the observed hospital-based and regional differences in KAPPr scores and APR are not obvious from our study data, especially as there were no remarkable differences in the implementation of AMS between hospitals surveyed.

Variabilities between hospitals in APR and knowledge of AMR have also been reported by various other studies from other parts of the world [55, 56] and they are usually fueled by contextual, cultural and behavioural factors that define the various hospitals [57]. Differences in the burden, types, and resistance patterns of ID between hospitals could determine the frequency and type of antibiotic prescribed. Organization culture may also influence how physicians prescribe [58]. Studies suggest that “prescribing etiquette” are usually created by senior physicians and passed on to junior physicians as part of mentorship process and to maintain the ‘culture’ of prescription within clinical groups [48, 59, 60]. Recognizing the importance of institutional context and peculiarities in the implementation of ASP, the WHO recommends institutional situational analysis and needs assessment before implementation of AMS in healthcare settings [16].

Our study data show that physicians practicing in paediatric department prescribed antibiotics more frequently than those from other departments, and physicians practicing in internal medicine and paediatric were more likely to prescribe the Reserve antibiotic Cefepime than those in other departments. Several PPS suggest that paediatric departments rank second only to the intensive care unit with respect to antibiotic use in hospital settings [38–41, 54]. These PPS have shown that APR are related to burden and resistance spectrum of bacterial infections among in-patients. Consequently, the observed variabilities of APR in relation to department of practice in our study could partly be due to higher burden of bacterial infections and AMR among patients in these departments. It could also be due to ‘prescription etiquette’ that favour more frequent APR in these departments.

Our study had several limitations. First, we did not evaluate all aspects of AMS in healthcare facilities as defined by the WHO [16]. However, our results identified fundamental gaps in the implementation of AMS in Nigeria tertiary hospitals deserving corrective interventions. Second, we did not use probability sampling in selecting respondents, and we could only enroll a limited number of respondents from the north-east region of the country due to prevailing security challenges in this region. However, our study is the first on KAP of AMR to include participants from all six geopolitical zones of the country. In view of the large sample size including participants from most parts of the country, we believe our sample is largely representative of the Nigeria context. Third, the frequency of antibiotic prescription was based on a qualitative Likert’s scale which may not necessarily reflect the quantitative equivalent of antibiotic prescription among respondents. Fourth, our study was not specifically designed to evaluate predictors of institutional or regional variabilities in good KAPPr. Future studies are necessary to determine other relevant contextual, cultural and behavioural factors that could influence KAP of antibiotic prescriptions among physicians in Nigeria.

In conclusion, our study results suggest a lack of implementation of AMS among tertiary hospitals in Nigeria. Although most physicians working in these hospitals had above average KAP of APR and AMR, there were gaps in knowledge and attitude, as well as frequent and inappropriate prescriptions of antibiotics, including those in the Reserve group. Good KAPPr were associated with institutional, geographical, educational, and professional factors. Our study findings should inform interventions that optimize the use of antibiotics in healthcare facilities and improve awareness and understanding of AMR in Nigeria in line with Nigeria’s NAPAR.

## Data Availability

The datasets in the present study are accessible from the corresponding author on reasonable request.

## Abbreviations

AMR: Antimicrobial resistance
AMRO: Antimicrobial resistant organisms
APR: Antibiotic prescription rate
ASP: Antibiotic stewardship programme
AMS: Antimicrobial stewardship
AWaRe: Access, Watch and Reserve categories
FCT: Federal Capital Territory
GAP: Global Action Plan
KAPPr: Knowledge, attitude, practice, and prescription
NAPAR: National action plan on antimicrobial resistance
PPS: Point prevalence surveys
Pr: Prescription
WHA: World Health Assembly
WHO: World Health Organization
